# The Challenges of Chromosome Y Analysis and the Implications for Chronic Kidney Disease

**DOI:** 10.3389/fgene.2019.00781

**Published:** 2019-09-04

**Authors:** Kerry Anderson, Marisa Cañadas-Garre, Robyn Chambers, Alexander Peter Maxwell, Amy Jayne McKnight

**Affiliations:** ^1^Epidemiology and Public Health Research Group, Centre for Public Health, Queen’s University of Belfast, c/o Regional Genetics Centre, Belfast City Hospital, Belfast, United Kingdom; ^2^Regional Nephrology Unit, Belfast City Hospital, Belfast, United Kingdom

**Keywords:** chromosome Y, chronic kidney disease, genome-wide association, genotyping arrays, haplogroup, imputation, LOY, microdeletion

## Abstract

The role of chromosome Y in chronic kidney disease (CKD) remains unknown, as chromosome Y is typically excluded from genetic analysis in CKD. The complex, sex-specific presentation of CKD could be influenced by chromosome Y genetic variation, but there is limited published research available to confirm or reject this hypothesis. Although traditionally thought to be associated with male-specific disease, evidence linking chromosome Y genetic variation to common complex disorders highlights a potential gap in CKD research. Chromosome Y variation has been associated with cardiovascular disease, a condition closely linked to CKD and one with a very similar sexual dimorphism. Relatively few sources of genetic variation in chromosome Y have been examined in CKD. The association between chromosome Y aneuploidy and CKD has never been explored comprehensively, while analyses of microdeletions, copy number variation, and single-nucleotide polymorphisms in CKD have been largely limited to the autosomes or chromosome X. In many studies, it is unclear whether the analyses excluded chromosome Y or simply did not report negative results. Lack of imputation, poor cross-study comparability, and requirement for separate or additional analyses in comparison with autosomal chromosomes means that chromosome Y is under-investigated in the context of CKD. Limitations in genotyping arrays could be overcome through use of whole-chromosome sequencing of chromosome Y that may allow analysis of many different types of genetic variation across the chromosome to determine if chromosome Y genetic variation is associated with CKD.

## Introduction

To date, the contribution of chromosome Y to the development and progression of chronic kidney disease (CKD) has remained largely unexplored. Over 50 genome-wide association studies (GWASs) have been conducted in renal diseases during the last 10 years (MacArthur et al., 2017), yet only one has reported details of chromosome Y analysis (Nanayakkara et al., 2014). For example, one of the most comprehensive meta-analysis GWASs conducted in renal disease included over 2.5 million single-nucleotide polymorphisms (SNPs), genotyped in 110,517 individuals; however, no chromosome Y SNPs were included (Gorski et al., 2017). The exclusion of chromosome Y from genomic analyses may previously have been justifiable, based on the assumption that chromosome Y was a “genetic wasteland” (Maan et al., 2017); but as more research is published, it is becoming clear that chromosome Y variation may be useful for identifying individuals with increased susceptibility to the disease. It was traditionally thought that chromosome Y only carried genes important for male-specific traits. However, pseudoautosomal regions (PARs) of sequence homology with chromosome X are found on the tips of chromosome Y. Gene expression levels between chromosome X and chromosome Y PAR homologs can be subject to male expression bias, whereby chromosome Y PAR genes are more highly expressed than their chromosome X counterparts, which could account for sex differences in disease (Snell and Turner, 2018). Additionally, upon the complete sequencing and characterization of the chromosome by Skaletsky et al. (2003), it was revealed that approximately 50% of protein-coding genes present on the male-specific region (MSY) expressed in non-gonadal tissues (Skaletsky et al., 2003), and it is, therefore, likely that they could play a role in common complex disease. For example, Charchar et al. (2012) performed such analysis on chromosome Y and identified an increased risk of coronary artery disease (CAD) in men from haplogroup I, but it is unclear whether this finding is relevant to CKD. The major rationale for including chromosome Y in studies of disease risk stems from the goal of identifying genetic features that may contribute to sex-specific presentations of disease. For example, cardiovascular disease (CVD) incidence is similar between men and women, but progression of the disease differs, with age of onset approximately 10 years later for women (De Smedt et al., 2016). Chromosome Y analysis to detect variation contributing to disease risk is a logical step. A very similar sexual dimorphism exists in CKD; prevalence is greater in women, but kidney disease in men progresses more rapidly to end-stage renal disease (ESRD), the most severe form of CKD (Hill et al., 2016). In this case, a male-specific marker of accelerated CKD progression could prove useful in identifying which patients are at greater risk of rapid loss of renal function. The issue of having better markers for CKD progression is relevant when examining current biomarkers for the diagnosis of CKD. Renal function is assessed by measuring either serum creatinine, or less commonly cystatin C, and an equation is used to determine an estimated glomerular filtration rate (eGFR). However, these eGFR equations are less accurate for certain individuals, such as those with low muscle mass, extreme body mass indexes, and early-stage CKD, a group whose identification is key to allow implementation of preventative measures (Gentile and Remuzzi, 2016). Extensive reviews of the literature have highlighted that there are relatively few alternative kidney function biomarkers, and none have improved upon the limitations of serum creatinine or cystatin C (Cañadas-Garre et al., 2018; Cañadas-Garre et al., 2019b). Therefore, while exclusion of chromosome Y in genomic analysis of renal disease may previously have been justifiable, it does highlight a distinct gap in our knowledge of how chromosome Y genetic variation may play a role in renal disease pathogenesis.

## Chromosome Y in Disease

While chromosome Y variation has been linked to a number of male-specific conditions such as prostate cancer (PCa), it has also been shown to influence the risk profile of men for common complex disease such as CAD and influence the progression of HIV (Sezgin et al., 2009). The clearest link thus far between chromosome Y and disease is related to infertility.

### Infertility

While up to 7% of men are infertile, only 15–30% of these cases have a known genetic cause (Neto et al., 2016). Numerical and structural defects in chromosome Y have been linked to male infertility. An extra copy of chromosome Y (47, XYY) is the second most-frequent aneuploidy of the sex chromosomes, present in 1/1,000 men (Bardsley et al., 2013) and can result in a complete lack of spermatozoa production (azoospermia) or a severely low sperm count (oligospermia) (McLachlan and O’Bryan, 2010). Chromosome Y microdeletions (deletions less than 5 megabases in size) (Halder et al., 2013) have long been associated with infertility (Stuppia et al., 1997), and three azoospermia factor regions, AZFa, AZFb, and AZFc, have been identified on the long arm of chromosome Y (Vogt et al., 1996). The most clinically significant recurrent microdeletions see the complete loss of each AZF region, or the combined loss of AZFb and AZFc, with approximately 80% of all microdeletions being a complete loss of AZFc (Krausz et al., 2014). The AZFc region, which contains the deleted in azoospermia (DAZ) gene family, is completely deleted in 5–10% of azoospermia/severe oligospermia cases, making it the most frequent genetic cause of infertility in men (Ferlin et al., 2005). Partial AZF deletions (Lu et al., 2009) and gr/gr deletions (Bansal et al., 2016) are associated with spermatogenic failure and have also been associated with different chromosome Y haplogroups (Lu et al., 2009; Ran et al., 2013; Xue et al., 2013). Copy number variation (CNV) in certain chromosome Y genes, such as *GOLGA2P3Y* and *RBMY1*, has been associated with reduced sperm count (Sen et al., 2016) and motility (Yan et al., 2017). However, while certain genes and deletions have been associated with male infertility, further research is required to establish a complete pathogenic mechanism.

### Prostate Cancer

Another male-specific condition with which chromosome Y has been linked is PCa. Loss of chromosome Y (LOY) has been observed in PCa (Noveski et al., 2016; Zhou et al., 2016). Specific deletions in chromosome Y genes have been associated with PCa, several of which, including the sex-determining factor *SRY*, were found to increase in frequency with increasing PCa stage (Perinchery et al., 2000). A similar study detected loss of the region containing *SRY* at a similar rate, and also observed this loss in surrounding benign prostate hyperplasia tissue, perhaps indicating that loss of *SRY* is a precursor for PCa (Jordan et al., 2001). Loss of *SRY* may prevent the negative regulation of the androgen receptor AR, leading to increased androgen receptor activity and thus PCa growth. An additional chromosome Y gene, *KDM5D*, is also thought to interact with the androgen receptor, altering the sensitivity of docetaxel, a drug commonly used in androgen deprivation therapy (Komura et al., 2016). Chromosome Y haplogroups (Paracchini et al., 2003), short tandem repeats (Carvalho et al., 2010; Nargesi et al., 2011), and a number of different genes, many identified through co-expression networks, have been associated with PCa (Khosravi et al., 2014). As the exact mechanism of PCa pathogenesis has yet to be elucidated, a further role of chromosome Y in PCa may yet emerge.

### Cardiovascular Disease

CVD and its associated conditions are an example of some of the strongest evidence linking chromosome Y to common complex disease. CVD is a prime example of a condition that exhibits a complex sexual dimorphism; incidence of CVD is higher in men than in age-matched women, but the relative risk of mortality is higher in women with CVD (Möller-Leimkühler, 2007). Chromosome Y genetic variation has previously been linked to CVD; carriers of haplogroup I-defining SNP rs2032597 (also known as M170) had a ∼50% higher age-adjusted risk of CAD than men with other chromosome Y lineages in two independent cohorts, and the joint analysis of both cohorts (Charchar et al., 2012). The presence of the A form of two SNPs, rs768983 (A/G) in *TBL1Y* and rs3212292 (A/T) in *USP9Y*, was associated with lower levels of triglycerides and higher levels of high-density lipoprotein (HDL)-cholesterol compared with the other haplotypes in Black individuals of African origin (Russo et al., 2008). These SNPs were almost entirely monomorphic in the other ethnic groups included in the analysis, highlighting the differences in risk profiles between different ethnic groups and may explain the lower risk of CVD in Black individuals. However, conflicting studies in this area make the connection between chromosome Y and CVD less clear. While the YAP polymorphism, caused by an Alu insertion, was associated with an increased risk of atherosclerotic plaque formation at a particular bifurcations (Voskarides et al., 2014), other studies failed to find any association between this polymorphism and low-density lipoprotein (LDL)-cholesterol (Shoji et al., 2002; Hiura et al., 2008), hypertension (Hiura et al., 2008; Kostrzewa et al., 2013), or myocardial infarction (MI) (Hiura et al., 2008). Other chromosome Y haplogroups have been investigated in CVD, and haplogroup K was found to be associated with a 2.5× increased risk of atherosclerotic plaque development (Hiura et al., 2008), but not with hypertension (Kostrzewa et al., 2013). Additional haplogroup analyses failed to identify any association between haplogroups and either hypertension (Kostrzewa et al., 2013) or venous thrombosis (de Haan et al., 2016). Conflicting evidence of association between the *Hin*dIII(±) polymorphism has also been presented, where *Hin*dIII(+) has been associated with increased systolic and diastolic blood pressure (Charchar et al., 2002) and MI in hypertensive patients (García et al., 2003). However, *Hin*dIII(−) has also been reported as associated with increased blood pressure, although this study was conducted in pre-pubescent boys, which may account for the conflicting results (Shankar et al., 2007). In other studies, no significant association at all was found between the *Hin*dIII(±) polymorphism and blood pressure (Rodriguez et al., 2005; Russo et al., 2006; Kostrzewa et al., 2013).

## Chromosome Y in Chronic kidney Disease

The potential links between chromosome Y genetic variation and CKD have not been systematically explored. Several types of genetic variation in chromosome Y have been discussed above in relation to disease: loss of chromosome Y (LOY), an extra copy of chromosome Y (47, XYY), chromosome Y microdeletions, CNVs, SNPs, and haplogroups. While many of these genetic variations, particularly microdeletions, CNVs, and SNPs, have been explored in autosomes in individuals with CKD, studies of these variants in chromosome Y in CKD are limited. LOY has been detected in other renal conditions, such as in renal cell carcinoma tumors (Dagher et al., 2013) but not in any condition falling under the umbrella of CKD. As a minimally invasive biomarker, LOY in CKD could be detected through traditional karyotyping or by using SNP arrays (Forsberg, 2017). No study to date has tested for association between mosaic LOY in peripheral blood and CKD; and as mosaic LOY in blood is also associated with smoking (Dumanski et al., 2015), higher cancer risk (Forsberg et al., 2014), aging (Forsberg et al., 2017), and age-related macular degeneration (Grassmann et al., 2019), any association study would require adjustment for these factors. An additional copy of chromosome Y results in a mild syndrome known as 47, XYY. CKD is not typically associated with 47, XYY, although a single participant with posterior urethral valves carrying an additional copy of chromosome Y was identified in a study of congenital anomalies of the kidney and urinary tract (CAKUT) (Caruana et al., 2014). Although 47, XYY does not appear to be associated with adult-onset CKD, its relevance in CKD has not really been explored, so the evidence is limited. As with LOY, XYY can be detected using karyotyping or SNP arrays. There are currently no known associations between chromosome Y microdeletions and CKD, as studies in CKD to date have generally focused only on the specific microdeletions described in autosomes. Microdeletions in the *HNF1B* gene on chromosome 17q12 have been identified in both children and adults with CKD (Musetti et al., 2014; Verbitsky et al., 2015). A study of idiopathic CKD found a 1.3Mb deletion in *HNF1B* in 9% of participants tested (Clissold et al., 2018). A microdeletion at 11p13, the region containing the *PAX6* and *WT1* genes, results in Wilms tumor, aniridia, genitourinary anomalies, and mental retardation (WAGR) syndrome, patients with which have, among other symptoms, significant ESRD (van Heyningen et al., 2007). Larger deletions and duplications, known as CNVs, have been studied in CKD but largely in pediatric cohorts of CAKUT. A study of two distinct cohorts of adult Han Chinese individuals found several CNVs in the *DEFA1A3* locus associated with renal dysfunction in immunoglobulin A nephropathy (IgAN) patients (Ai et al., 2016). *NPHP1* gene deletions resulting in nephronophthisis, one of the most prevalent causes of ESRD in children, have also been detected in patients with adult-onset ESRD (Snoek et al., 2018). Among these studies, different CNV detection methods were used, including SNP arrays and whole-exome sequencing (Caruana et al., 2014; Bekheirnia et al., 2017). While some studies actively did not analyze any of the known chromosome Y CNVs (Verbitsky et al., 2015; Li et al., 2017), whether chromosome Y was included in the analysis in other studies was unclear. Therefore, due to either lack of reported analysis or indeed lack of association, there are currently no known associations between chromosome Y CNVs and CKD.

As outlined above, more than 50 studies have tried to unravel the genetic variation of CKD explained by SNPs (MacArthur et al., 2017). To date, 140 autosomal and X chromosome SNPs have been associated with CKD (Cañadas-Garre et al., 2019a). However, no studies have reported significant associations with chromosome Y SNPs, probably because they were methodologically excluded. Indeed, only one GWAS of renal dysfunction appears to have included chromosome Y SNPs in their analysis (Nanayakkara et al., 2014). No significant associations were detected between CKD and chromosome Y SNPs in this study, but due to differences in prevalence of chromosome Y haplogroups between different populations, this does not necessarily mean chromosome Y variation is not linked to CKD. For example, the most common haplogroup in European populations, R1b, is not present in the Sinhalese participants of the study by Nanayakkara and colleagues, whose dominant haplogroup is the R2 haplogroup (Kivisild et al., 2003), therefore demonstrating the need for analysis in a range of populations. Given that chromosome Y SNPs have demonstrated to play a role in other diseases, particularly CVD, a condition with strong links to CKD, are we missing associations between chromosome Y SNPs and CKD?

## GWAS Exclusion

**Figure 1:**
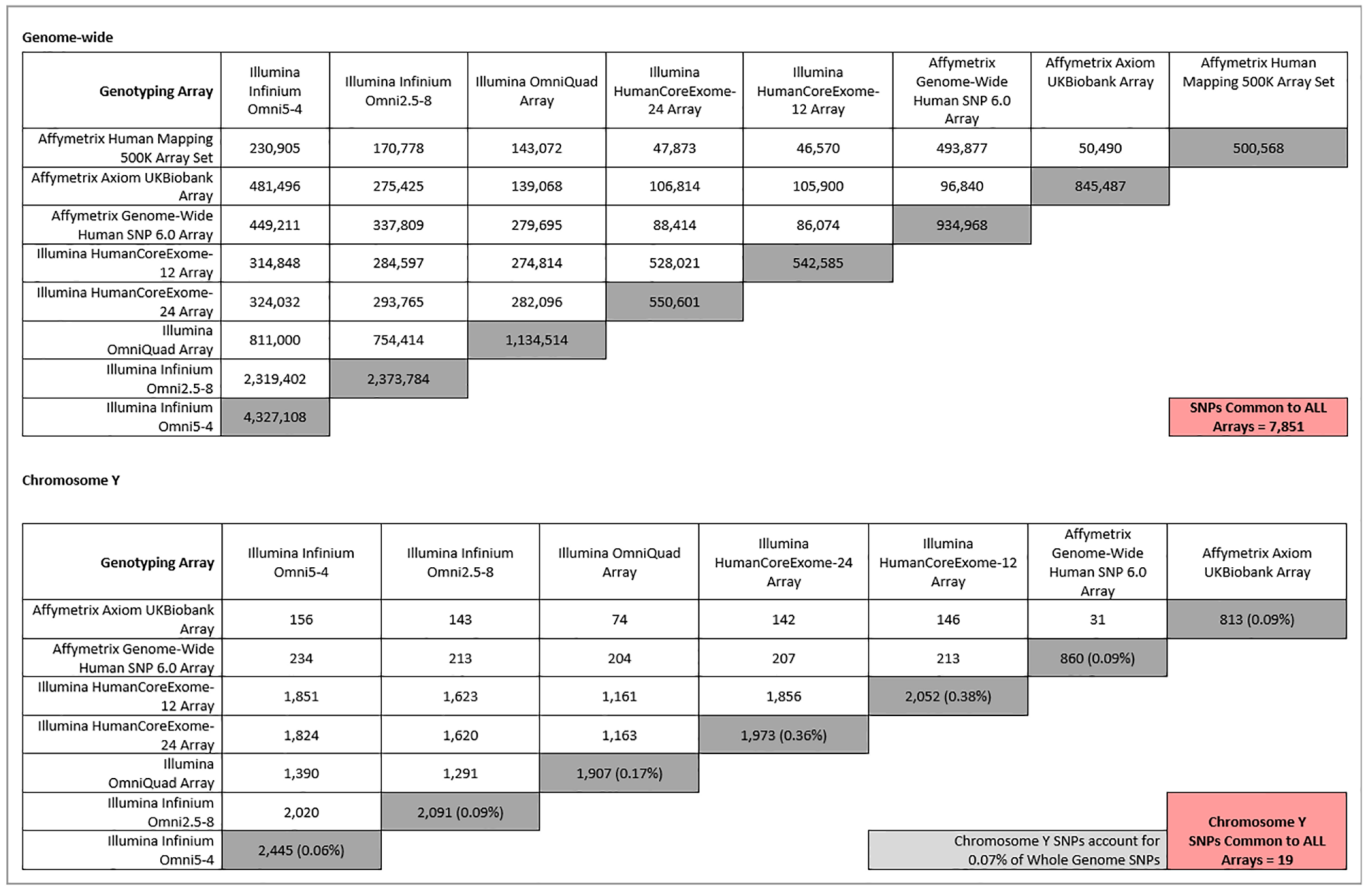
Comparison of whole-genome (top) and chromosome Y (bottom) SNPs between different commonly used genotyping platforms. Dark grey-shaded boxes indicate the total number of either whole-genome or chromosome Y SNPs present on each array. The percentages that chromosome Y SNPs make up of their respective arrays are shown in brackets in the dark grey-shaded boxes. The light grey-shaded box is the percentage chromosome Y SNPs in the entire genome, for comparison. Pink-shaded boxes show the number of SNPs common between all arrays for either whole genome (top) or chromosome Y SNPs (bottom). The Affymetrix 500K array has been excluded from the chromosome Y section (bottom), as it contains zero chromosome Y SNPs.

**Figure 2:**
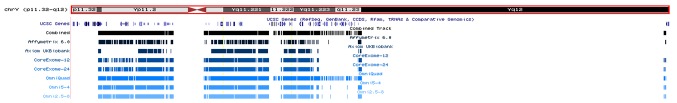
Positions of chromosome Y SNPs on each array in relation to chromosome Y genes from the UCSC database (Kent et al., 2002). “Combined” track contains all SNPs from the six individual array tracks (*n* SNPs = 4344). Chromosome Y SNPs from the pseudoautosomal regions are not included here, explaining the lack of gene coverage at the start and the end of the chromosome.

Chromosome Y SNPs make up approximately 0.07% (60,505/84,387,209) of all recorded biallelic SNPs within the genome (Gibbs et al., 2015). Therefore, a possible explanation for the lack of significant findings on chromosome Y in relation to CKD may be the underrepresentation of chromosome Y on commonly used genotyping arrays. **Figure 1** shows that, although chromosome Y is completely excluded from some arrays, its representation on other platforms is actually greater than the percentage of chromosome Y SNPs in the genome. However, although chromosome Y may be represented almost proportionally on genotyping arrays, only 4% of chromosome Y SNPs in the genome (60,555) are analyzed on the largest array (2,445 on the Illumina Omni-5.4 Array). Therefore, while representation of chromosome Y on traditional genotyping arrays may be *proportional*, it is far from comprehensive. Insufficient gene coverage may also explain the lack of significant findings in chromosome Y. However, as shown in **Figure 2**, the SNPs offered on commonly used genotyping arrays provide sufficient coverage of chromosome Y genes (Zerbino et al., 2018).

Although only a fraction of chromosome Y SNPs are present on available genotyping platforms, an often greater-than-proportional number of SNPs is dedicated to chromosome Y on common genotyping platforms. So why is chromosome Y excluded from analyses? Chromosome Y content between genotyping arrays is variable, which in turn greatly limits the number of SNPs available for cross-study meta-analysis. For example, for a meta-GWAS of cohorts genotyped using the arrays outlined in **Figure 1**, no chromosome Y SNPs would be eligible for inclusion, as zero chromosome Y SNPs are found on the Affymetrix 500K arrays. This may explain why chromosome Y is excluded from some larger meta-GWAS. Even if arrays do contain chromosome Y SNPs, there is a distinct lack of overlap between arrays, and as more arrays are considered, the number of SNPs that are common to all platforms is reduced. For example, **Figure 1** shows that only 19 SNPs are in common between the other seven included arrays.

However, the same can be said for the rest of the SNPs on commonly used genotyping platforms. Whole-genome SNPs (including chromosome Y) that are common between the same eight genotyping arrays are outlined in **Figure 1**. Only 7,851 SNPs feature on all eight arrays. Yet in larger renal meta-GWAS, by using imputation, as many as 2.5 million autosomal and chromosome X SNPs can be included (Pattaro et al., 2016; Gorski et al., 2017). Imputation is a process that allows inference of ungenotyped SNPs in a sample, based on panels of haploid reference sequences (Marchini et al., 2007), meaning that the number of loci for which information is available can be dramatically increased from the number obtained from direct genotyping alone. For example, less than a million directly typed markers were imputed to approximately 96 million variants using the Human Reference Consortium and UK10K haplotype resources in UK Biobank samples genotyped using either the UK BiLEVE or UK Biobank Axiom array (Bycroft et al., 2018). In this case, imputation allowed more than a hundred times the number of directly genotyped SNPs to be available for analysis. Even after applying quality control thresholds, such as for minor allele frequency and imputation quality, as many as 12 million SNPs could be available for association analysis (Haas et al., 2018). The same imputed genotypes were used in a recent meta-GWAS, in which five million SNPs were common between the three studies and available for the meta-analysis (Xue et al., 2018).

However, as chromosome Y is haploid and a large portion of the chromosome does not undergo recombination, accurate and reliable chromosome Y imputation is, despite recent efforts (Zhang et al., 2013), not widely implemented. Even chromosome X, whose imputation has been achieved (Marchini and Howie, 2010), may be excluded due to the need to impute it separately, so exclusion of both sex chromosomes in GWAS is common. While this lack of recombination in the majority of chromosome Y should actually aid imputation, chromosome Y reference panels for imputation are not widely available, and therefore, chromosome Y is often excluded from the analysis at this stage. For example, the Sanger Imputation service offers five different reference panels to impute data to, none of which include chromosome Y (McCarthy et al., 2016). However, the lack of recombination across this section of chromosome Y means that any genetic variations in the MSY pass directly from father to son and means that certain genetic variations are often inherited together. Patterns in these genetic variations are known as haplotypes and can be used to group individuals into haplogroups. These groups or “clades” are defined by single markers that differentiate one clade from another, and genotyping of these markers can be used to sort individuals into different haplogroups. It has also been shown that some of these haplogroups are associated with certain phenotypes. For example, haplogroup I, one of the most frequently occurring haplogroup in the UK (Winney et al., 2012), is associated with an increased risk of CAD (Charchar et al., 2012). In many cases, haplotyping negates the need for large numbers of SNPs to be genotyped in genetic association analyses; for example, only 11 SNPs need to be genotyped to cover 95% of the haplogroups present within the UK (Charchar et al., 2012). Haplotyping can be performed using any SNPs genotyping method, including SNP arrays (Kim and Misra, 2007). All arrays included in **Figure 1** provide coverage of major haplogroup-defining SNPs and would therefore be suitable for use in haplogroup association studies. However, to date, no such analysis has been carried out in CKD. This glaring lack of investigation offers the opportunity to perform a complete analysis of chromosome Y genetic variation in CKD. A full analysis of SNPs/haplogroups, CNVs, microdeletions, LOY, and XYY could be performed using SNP arrays, but the limitations of these arrays in chromosome Y have been outlined above. The decreasing cost of and increased coverage offered by whole-genome (WGS) and whole-exome sequencing (WES) may offer a solution for improving investigations in chromosome Y (Levy and Myers, 2016).

## Whole Genome/Exome Sequencing and Chromosome Y

WGS has already been utilized in CKD. WES detected diagnostic variants (Lata et al., 2018; Groopman et al., 2019) and CNVs in individuals with CKD (Bekheirnia et al., 2017); chromosome Y was not included in either of these studies, as they only targeted variants with known links to CKD. WGS can also detect LOY/XYY, microdeletions, SNPs and therefore, haplogroups (Muzzey et al., 2015). It offers the added benefit of multiple long reads, meaning there is a reduced risk of genotypes being lost to poor genotyping, as there is with arrays. WGS would generate a complete profile of chromosome Y to be analyzed, rather than needing multiple alternative methods (karyotyping, chromosomal arrays, and SNP arrays) to provide the same data. As knowledge of chromosome Y haplogroups grows, it is expected that more haplogroup markers will be added to the phylogeny (van Oven et al., 2014) and that WGS prevents re-genotyping of samples for certain markers as the whole sequence will be available. WGS also overcomes the issue of imputation; genotypes do not need to be inferred if the whole sequence is available. Lack of common SNPs between different studies is also improved by access to the entire sequence. However, due to sequence homology between the sex chromosomes and the large regions of repetitive sequences within chromosome Y, sequencing presents some challenges in itself. Repetitive sequences make sequencing more difficult. However, tools are being developed to try and combat some of these issues (Webster et al., 2019), and as sequencing technologies develop and costs fall, longer reads of greater read depth may aid in mapping complex repeated sequences. For example, Oxford Nanopore technology has recently achieved read lengths of up to 2.2Mb (Payne et al., 2018), and this has since been used to sequence and assemble the first chromosome Y of African Origin, where sequence continuity increased by almost 800% than did previous methods and amounted to 21.5Mb of total sequence (Kuderna et al., 2019). In short, although presenting some challenges of its own, WGS/WES resolves many of the major issues of analyzing chromosome Y with SNP arrays and allows multiple types of variation to be considered using a single test, offering the most comprehensive analysis of chromosome Y possible.

## Conclusions

Chromosome Y analysis remains challenging due to lack of common coverage by genotyping arrays, the need to process chromosome Y data separately from autosomal data, and the current inability to accurately impute chromosome Y to the same standards that have been achieved in autosomal imputation. The inclusion of chromosome Y in GWAS and other genetic analyses is inconsistent, and in many cases, it is not clear if the analysis was not performed, or if negative results have simply not been reported. For this reason, the known contribution of chromosome Y genetic variation to disease remains limited, particularly in renal disease. The sexual dimorphism in CKD provides a rationale for further investigations of factors influencing sex-related progression to ESRD, perhaps using methods such as targeted next-generation sequencing to analyze chromosome Y specifically. While other factors, such as hormone profiles, may influence disease progression, the current lack of chromosome Y analysis in renal disease means that the contribution of genetic variation in chromosome Y to renal disease progression remains unknown.

## Author Contributions

KA: Conception and design of the work; acquisition, analysis, and interpretation of the data for the article; and drafting, critical revision, and final approval of the manuscript. MCG: Interpretation of the data for the article and drafting, critical revision, and final approval of the manuscript. RC: Acquisition, analysis, and interpretation of the data for the article and drafting and final approval of the manuscript. APM: Conception and design of the work; interpretation of the data for the article; and drafting, critical revision, and final approval of the manuscript. AJM: Conception and design of the work; interpretation of the data for the article; and drafting, critical revision, and final approval of the manuscript.

## Funding

This work has been partly funded by the Medical Research Council (Award reference MC_PC_15025) and the Public Health Agency R&D Division (Award Reference STL/4760/13). KA and MG are funded by a Science Foundation Ireland-Department for the Economy (SFI-DfE) Investigator Program Partnership Award (15/IA/3152).

## Conflict of Interest Statement

The authors declare that the research was conducted in the absence of any commercial or financial relationships that could be construed as a potential conflict of interest.

## Abbreviations

AZF, Azoospermia factor; CAD, Coronary artery disease; CAKUT, Congenital anomalies of the kidney and urinary tract; CKD, Chronic kidney disease; CNV, Copy number variation; CVD, Cardiovascular disease; DAZ, Deleted in azoospermia; eGFR, Estimated glomerular filtration rate; ESRD, End-stage renal disease; GWAS, Genome-wide association study; HDL, High-density lipoprotein; HIV, Human immunodeficiency virus; IgAN, Immunoglobulin A nephropathy; LDL, Low-density lipoprotein; LOY, Loss of chromosome Y; MI, Myocardial infarction; MSY, Male-specific region of chromosome Y; PAR, Pseudoautosomal region; PCa, Prostate cancer; SNP, Single-nucleotide polymorphism; WAGR, Wilms tumor, aniridia, genitourinary anomalies, and mental retardation; WES, Whole-exome sequencing; WGS, Whole-genome sequencing; YAP, Chromosome Y Alu insertion polymorphism.
